# Ultra-Wideband Optically Transparent Absorbing Metasurface Based on Multilayer ITO Films

**DOI:** 10.3390/nano16171110

**Published:** 2026-09-03

**Authors:** Guang Lu, Mingyang Liu, Bing Wang

**Affiliations:** 1 School of Space Science and Technology, Shandong University at Weihai, Weihai 264209, China; 2 Laboratory for ElectromAgnetic Detection (LEAD), Institute of Space Sciences, Shandong University, Weihai 264209, China

**Keywords:** metasurface, indium tin oxide (ITO), optical transparency, ultra-wideband absorption

## Abstract

Traditional microwave-absorbing metasurfaces struggle to integrate optical transparency with ultra-wideband and high-efficiency microwave absorption, severely limiting their deployment in optoelectronics-compatible electromagnetic protection. To address this constraint, we propose a transparent ultra-wideband microwave-absorbing metasurface based on multilayered indium tin oxide (ITO) films. The unit cell is constructed using a multilayered PMMA dielectric configuration, where ITO conductive layers are patterned as a top square patch, middle square rings, and a continuous bottom film. Full-wave parametric optimization, combined with multilayer resonant coupling, effectively extends the absorption bandwidth. We elucidate the underlying broadband absorption mechanism by analyzing electromagnetic field and surface current distributions at typical resonant frequencies. Furthermore, we systematically investigate the impacts of ITO sheet resistance, incident angle, and polarization state on absorption performance. A 6 × 6 array prototype is fabricated and experimentally characterized in a microwave anechoic chamber. The measured results demonstrate that the proposed metasurface achieves an absorptance exceeding 90% across 9.2–40.2 GHz, delivering a fractional bandwidth of 125.5%. The experimental responses are in good agreement with numerical simulations, and the fabricated prototype retains good optical transparency. Benefiting from the synergistic integration of ultra-wideband microwave absorption and superior optical transmissivity, this multilayer stacked metasurface offers a promising strategy for advanced optoelectronics-compatible stealth and transparent electromagnetic shielding applications.

## 1. Introduction

Recent advances in wireless communication, radar detection, and high-frequency electronics have intensified concerns over electromagnetic interference and radiation [1,2,3], threatening both precision equipment and human health [4,5]. Microwave absorbers and metasurfaces thus attract broad interest [6], yet conventional designs rely on opaque metal backings or thick magnetic composites, blocking light transmission [7]. This opacity prevents their deployment in scenarios where both electromagnetic protection and optical transmission are required, such as aircraft canopies, vehicle windshields, smart building windows and photovoltaic panels, making optical transparency a critical functional indicator rather than a redundant performance parameter [8]. Existing absorbing metasurfaces seldom achieve simultaneous optical transparency and ultra-wideband high-efficiency absorption, hindering optoelectronic compatible electromagnetic protection.

To achieve optically transparent microwave absorption, researchers have explored a variety of transparent conductive materials and structural designs [9,10]. Early transparent absorbers primarily utilized transparent conductive polymers, carbon-based nanomaterials, and metallic micromeshes [11,12]. Although metallic mesh can deliver high electrical conductivity and transparency, its fabrication process is intricate, and it suffers from oxidation and visual Moiré fringe effects [13,14]. Graphene exhibits excellent tunable electrical properties, but the large-area, low-cost uniform preparation remains a significant technical bottleneck [15,16,17,18]. In contrast, transparent conductive oxides (TCOs), and indium tin oxide (ITO) in particular, are widely recognized as promising materials for transparent absorbing metasurfaces. ITO benefits from well-established fabrication processes, favorable physicochemical stability, and high transmittance in the visible spectrum. At microwave frequencies, ITO behaves as a tunable resistive thin film, whose tailorable sheet resistance enables proper impedance matching with free space and sufficient ohmic loss to dissipate incident electromagnetic energy [19,20,21,22]. Currently, a wide variety of ITO-based optically transparent microwave absorbing metasurfaces has been investigated [23]. For instance, single-layer ITO patterned metasurfaces have been proposed; however, restricted by their single resonant mode, these ultra-thin structures typically exhibit a narrow absorption bandwidth, failing to meet the demand for broadband electromagnetic protection [24]. To broaden the operating bandwidth, researchers have introduced multilayer stacked architectures, cascading various ITO resonant layers with transparent dielectric spacers such as Polyethylene Terephthalate (PET) and Polymethyl Methacrylate (PMMA) [25].

This paper proposes an optically transparent ultra-wideband microwave absorbing metasurface based on multilayer ITO films to overcome the bandwidth-transmittance trade-off of conventional transparent absorbers. The unit cell adopts a PMMA dielectric multilayer stack, in which the ITO layers are patterned as a top square patch, intermediate square rings, and a bottom continuous film. Full-wave simulation optimization of structural parameters, combined with multilayer resonant coupling, significantly broadens the absorption bandwidth. This work realizes broadband electromagnetic regulation via microstructure engineering of ITO nanofilms, which represents a typical application of transparent conductive nanomaterials in microwave functional devices.

## 2. Design and Simulation of the Metasurface

**Figure 1:**
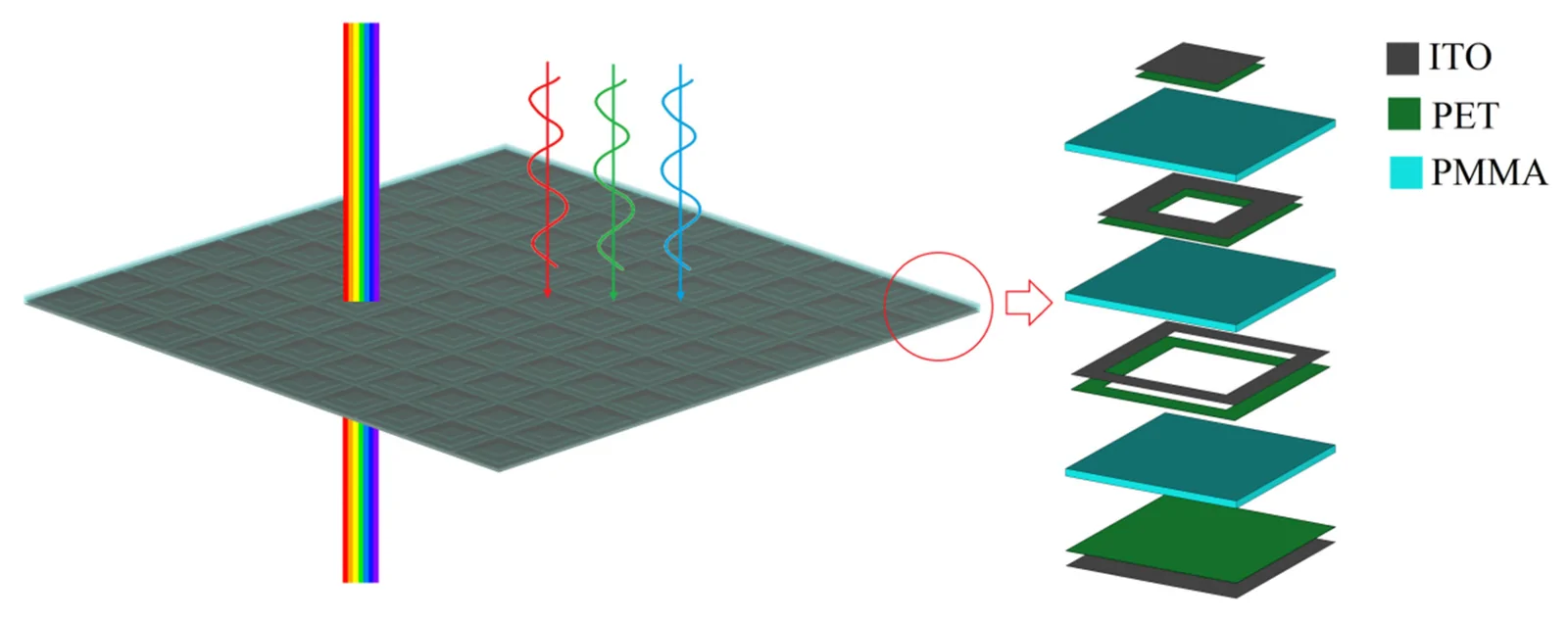
Schematic of the optically transparent broadband microwave-absorbing metasurface. The colored light columns represent incident visible light; the red, green, and blue curves with arrows denote the incident microwave signals. The layered structure on the right is the magnified view of the region in the circle.

We propose an optically transparent broadband microwave-absorbing metasurface utilizing multilayered indium tin oxide (ITO) films, as shown in Figure 1. The unit cell comprises three stacked transparent PMMA substrates. Each resonant ITO layer is deposited on a PET film, which is subsequently laminated onto the PMMA substrate surface. The top ITO layer is patterned into a square patch, the two intermediate layers into square rings, and the bottom layer forms a continuous conductive film. Upon normal incidence, electromagnetic waves at disparate frequencies trigger coupled resonances within the multilayered ITO architectures to realize broadband absorption. Meanwhile, the adopted PET, PMMA and ITO materials possess intrinsic optical transparency. All full-wave simulations were conducted in CST Microwave Studio (version 2017) [26]. For the infinite-periodic unit cell, unit-cell boundary conditions were applied in the X and Y directions, with two waveguide ports on Zmin and Zmax for Floquet-mode normal incidence. It should be noted that periodic structures can generate higher-order propagating Floquet diffracted modes above the diffraction cutoff frequency. Nevertheless, the Floquet waveguide ports only output S-parameters associated with zero-order specular reflection and forward transmission, which matches the measurement convention adopted in our experiments. The patterned ITO layers are modelled via the ohmic-sheet condition. The frequency-domain solver with tetrahedral mesh and adaptive refinement was adopted to ensure S-parameter convergence.

**Figure 2:**
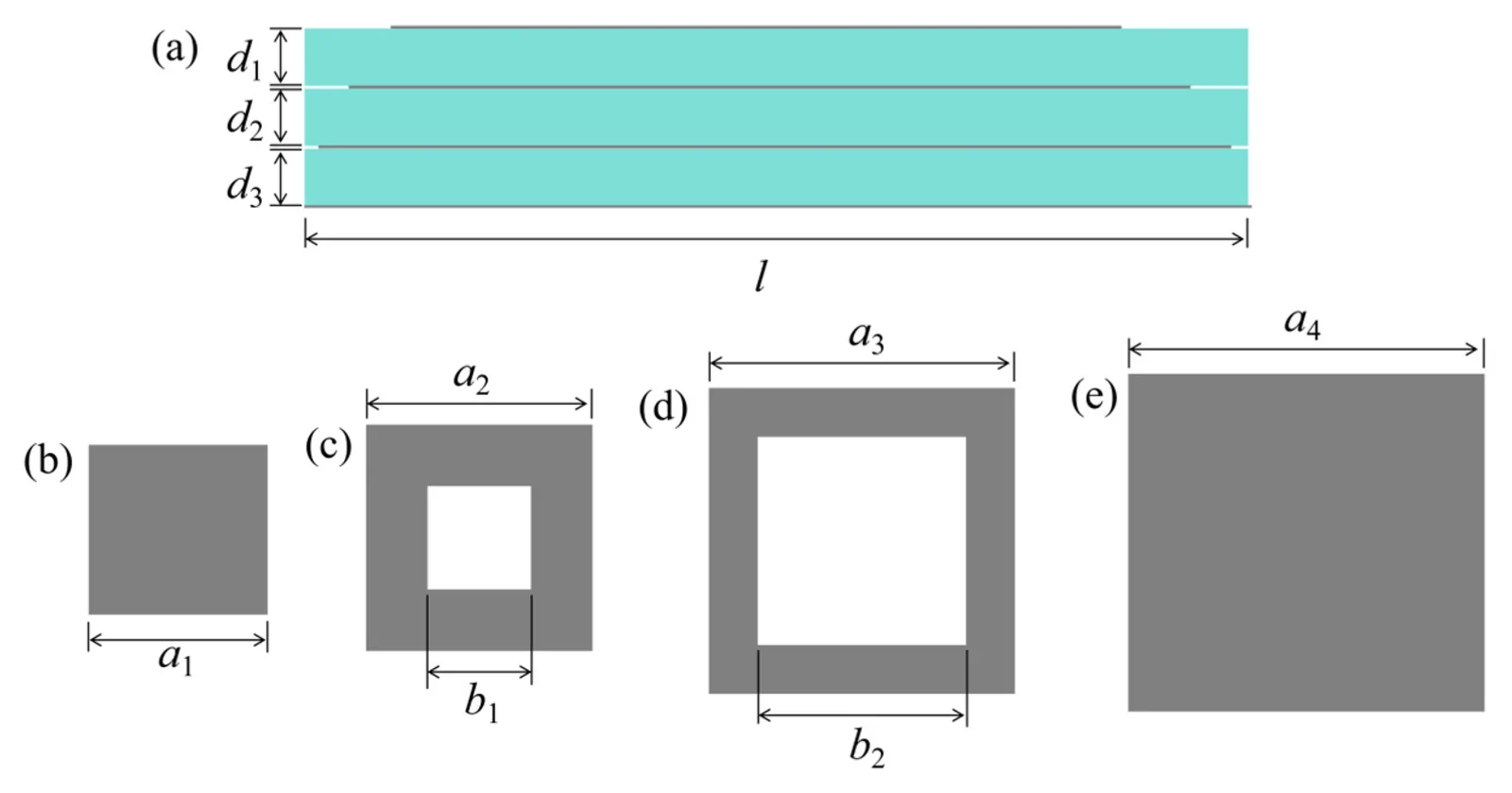
Schematic geometry of the metasurface unit cell: (**a**) cross-sectional view; (**b**) top ITO square patch; (**c**) second-layer ITO square ring; (**d**) third-layer ITO square ring; (**e**) bottom continuous ITO film.

Figure 2 details the geometric configuration and dimensional parameters of the proposed unit cell. As shown in Figure 2a, the cross-section along the thickness direction demonstrates that the dielectric components are PMMA slabs with a relative permittivity ε_PMMA_ = 2.25. The three PMMA layers possess the same thickness, *d*_1_ = *d*_2_ = *d*_3_ = 2 mm, and the unit cell periodicity is set as *l* = 50 mm. Figure 2b–e display the planar pattern of each resonant ITO layer, all implemented with a sheet resistance of 150 Ω/sq, which is physically valid as the film thickness is far smaller than the operating wavelength. PMMA and PET were treated as lossless dielectrics in simulation due to their extremely low intrinsic microwave loss. Parametric optimization was implemented via systematic parameter sweep to maximize the 90% absorption bandwidth, under the geometric constraint *a*_4_ > *a*_3_ > *a*_2_ > *b*_2_ > *a*_1_ > *b*_1_ (*a*_4_ = unit-cell period *l* = 50 mm). Optimal dimensions were obtained by sweeping the sizes of the top patch and intermediate rings within reasonable ranges. The top ITO layer is patterned into a square patch with side length *a*_1_ = 12 mm. The second layer forms a square ring with an outer edge length of 38 mm and an inner edge length *b*_1_ = 20 mm. The third layer adopts another square ring geometry with outer side length *a*_3_ = 48 mm and inner side length *b*_2_ = 36 mm. The bottom layer consists of a continuous ITO film with side length *a*_4_ = 50 mm, consistent with the unit cell period. All conductive ITO films are deposited on 0.125 mm-thick PET substrates, whose relative permittivity is *ε_PET_* = 3.0. The complete optically transparent microwave-absorbing metasurface is constructed by periodically arranging these unit cells.

**Figure 3:**
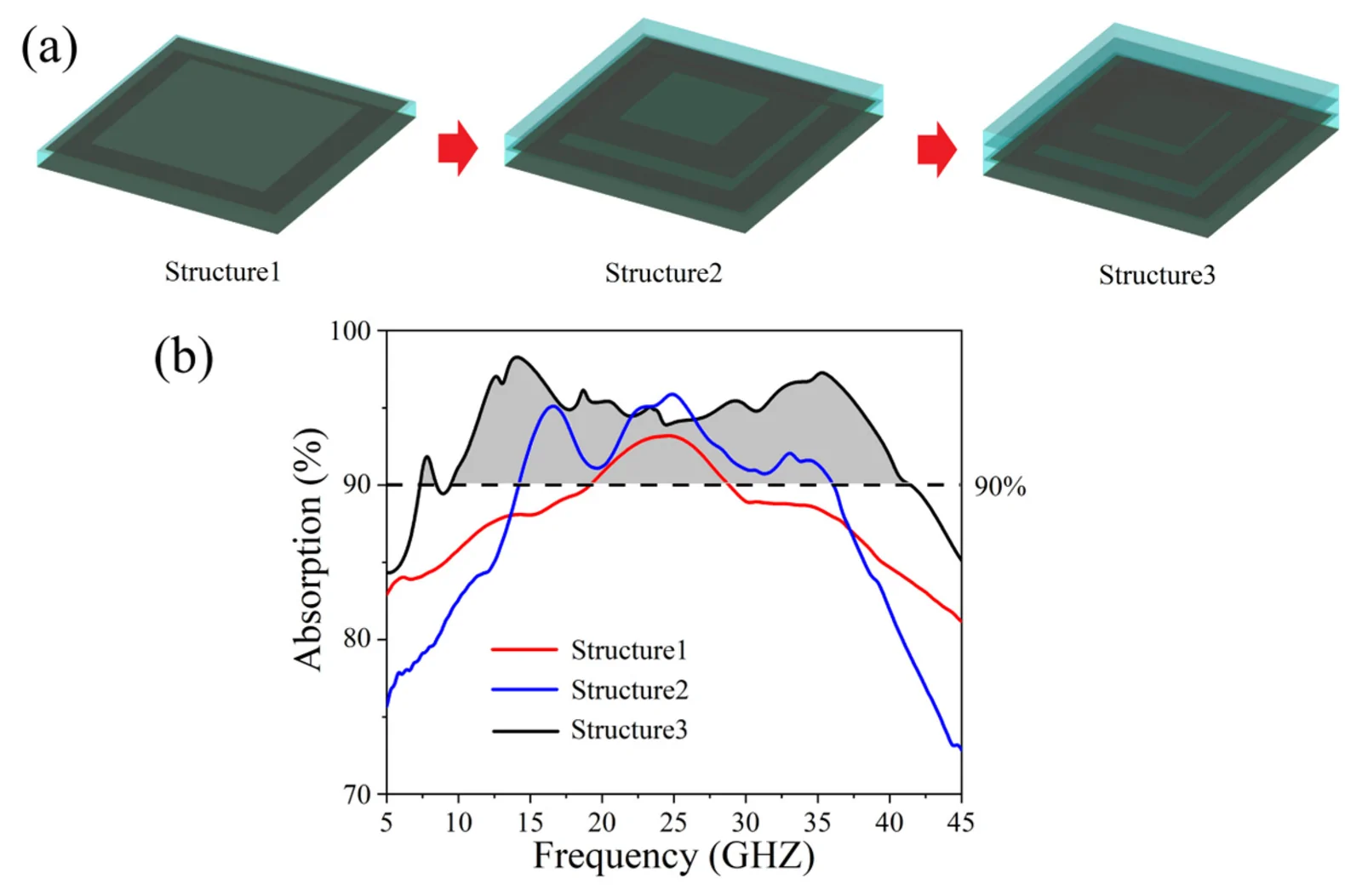
Structural evolution and simulated absorption performance of the metasurface: (**a**) schematic of three progressive configurations; (**b**) corresponding absorption spectra of Structures 1, 2, and 3.

To reveal the bandwidth broadening mechanism originating from multilayer resonant elements, we establish three evolutionary structural models in sequence, as sketched in Figure 3a. Structure 1 comprises one PMMA substrate integrated with two ITO resonant layers. Structure 2 is developed by adding an extra PMMA slab and one ITO resonant layer to Structure 1, forming a two-layer PMMA architecture equipped with three ITO resonant layers. Structure 3, the final proposed design, is further supplemented with a top ITO square patch to construct the multilayer stacked metasurface. Full-wave simulations are carried out within CST Microwave Studio, and the derived absorption spectra are presented in Figure 3b. Structure 1 achieves over 90% absorptance across 19.26–28.76 GHz. Structure 2 extends the effective absorption bandwidth to 14.22–36.10 GHz, verifying that multilayer resonant stacking is an effective strategy for bandwidth expansion. After further optimization, Structure 3 realizes >90% absorptance from 9.50 to 41.30 GHz, delivering an effective absorption bandwidth of 31.80 GHz and a fractional bandwidth of 125.2%. These simulated results verify that the proposed multilayer coupling configuration is capable of realizing ultra-wideband microwave-absorbing performance.

**Figure 4:**
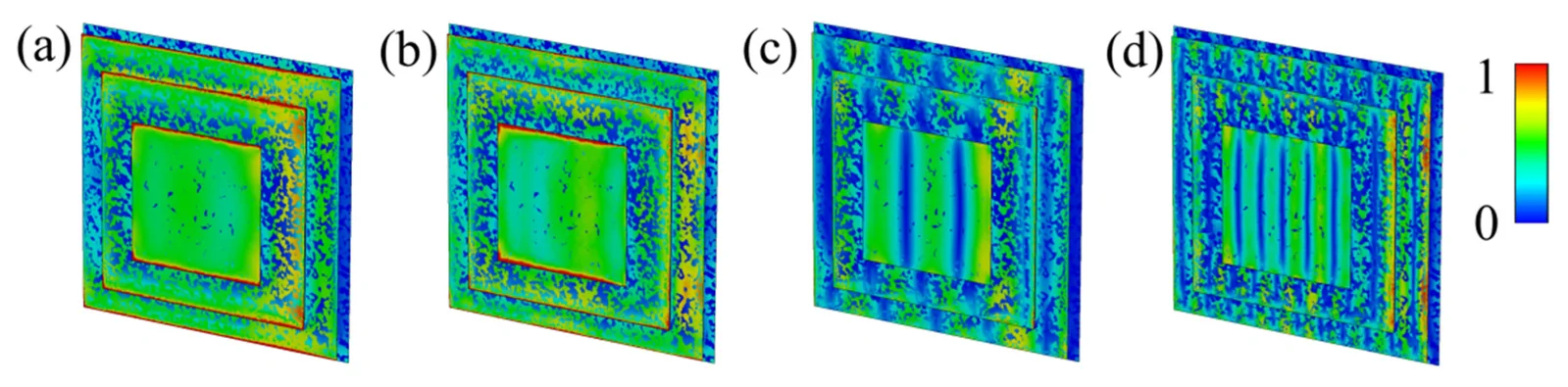
Simulated electromagnetic field distributions within the unit cell at characteristic frequencies: (**a**) electric field at 14.0 GHz; (**b**) electric field at 35.2 GHz; (**c**) magnetic field at 14.0 GHz; (**d**) magnetic field at 35.2 GHz.

To uncover the intrinsic mechanism, we analyze the electromagnetic field distributions at 14.0 and 35.2 GHz within the absorption band, as shown in Figure 4. At 14.0 GHz, the electric field concentrates along the edges of ITO patches and rings, with clear interlayer coupling, while the magnetic field is uniformly distributed across the dielectrics, indicating joint participation of all layers in the low-frequency resonance. At 35.2 GHz, the electric field remains edge-confined but the resonance mode shifts, and the magnetic field exhibits a standing-wave pattern. Distinct modes excited at different frequencies enable the multilayered ITO resonators to respond across separate bands; their superposition and coupling expand the high-efficiency bandwidth.

**Figure 5:**
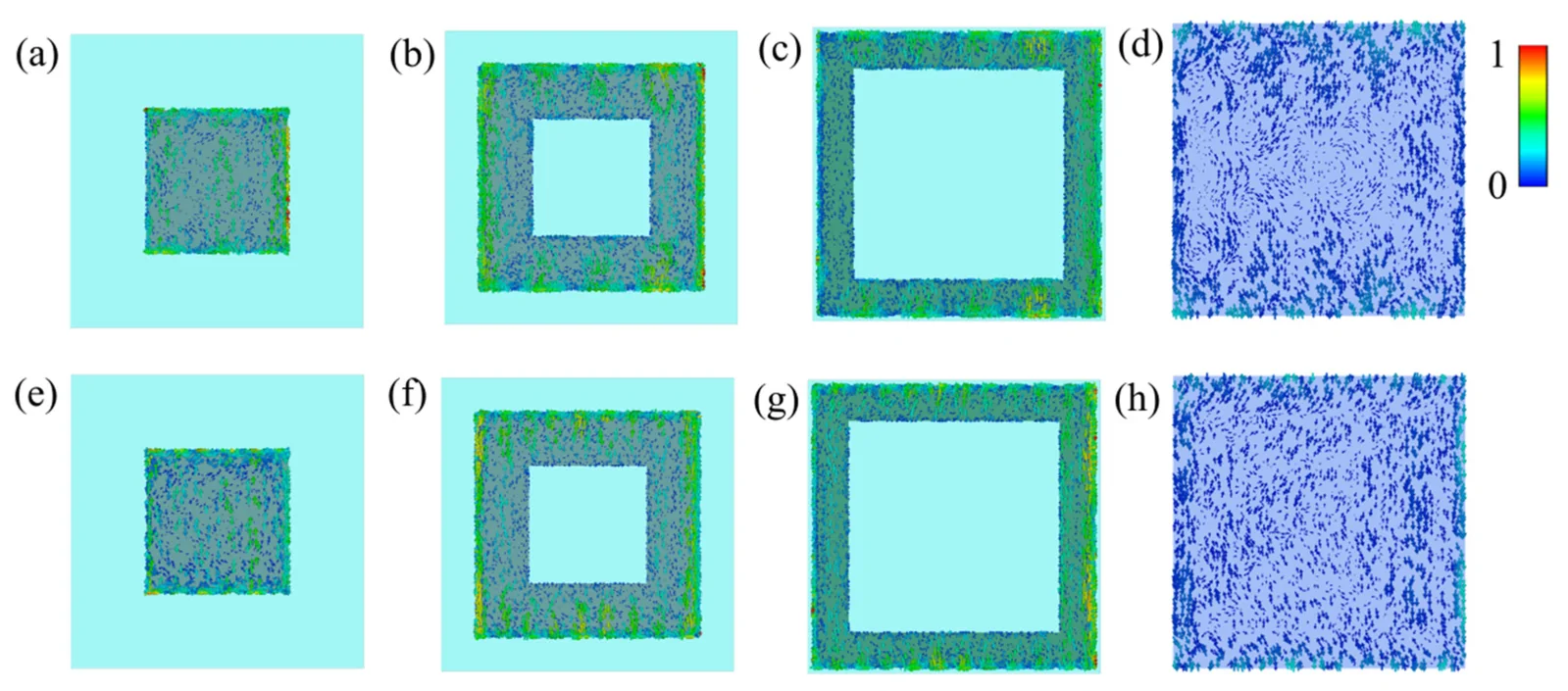
Surface current distributions on each ITO layer at characteristic frequencies: (**a**–**d**) 14.0 GHz; (**e**–**h**) 35.2 GHz; (**a**,**e**) top square patch, (**b**,**f**) second-layer square ring, (**c**,**g**) third-layer square ring, (**d**,**h**) bottom continuous ITO film.

Surface current distributions (Figure 5) further clarify the role of each ITO layer. At 14.0 GHz, strong currents appear on the top patch, both rings, and the bottom film, confirming that all four layers contribute with strong interlayer coupling. At 35.2 GHz, currents persist on the top and ring layers but weaken on the bottom film, indicating that the upper three layers dominate the high-frequency resonance. The overlap and coupling of these modes, supported by different layer combinations, sustain efficient loss over a broad range, thus achieving ultra-wideband absorption.

**Figure 6:**
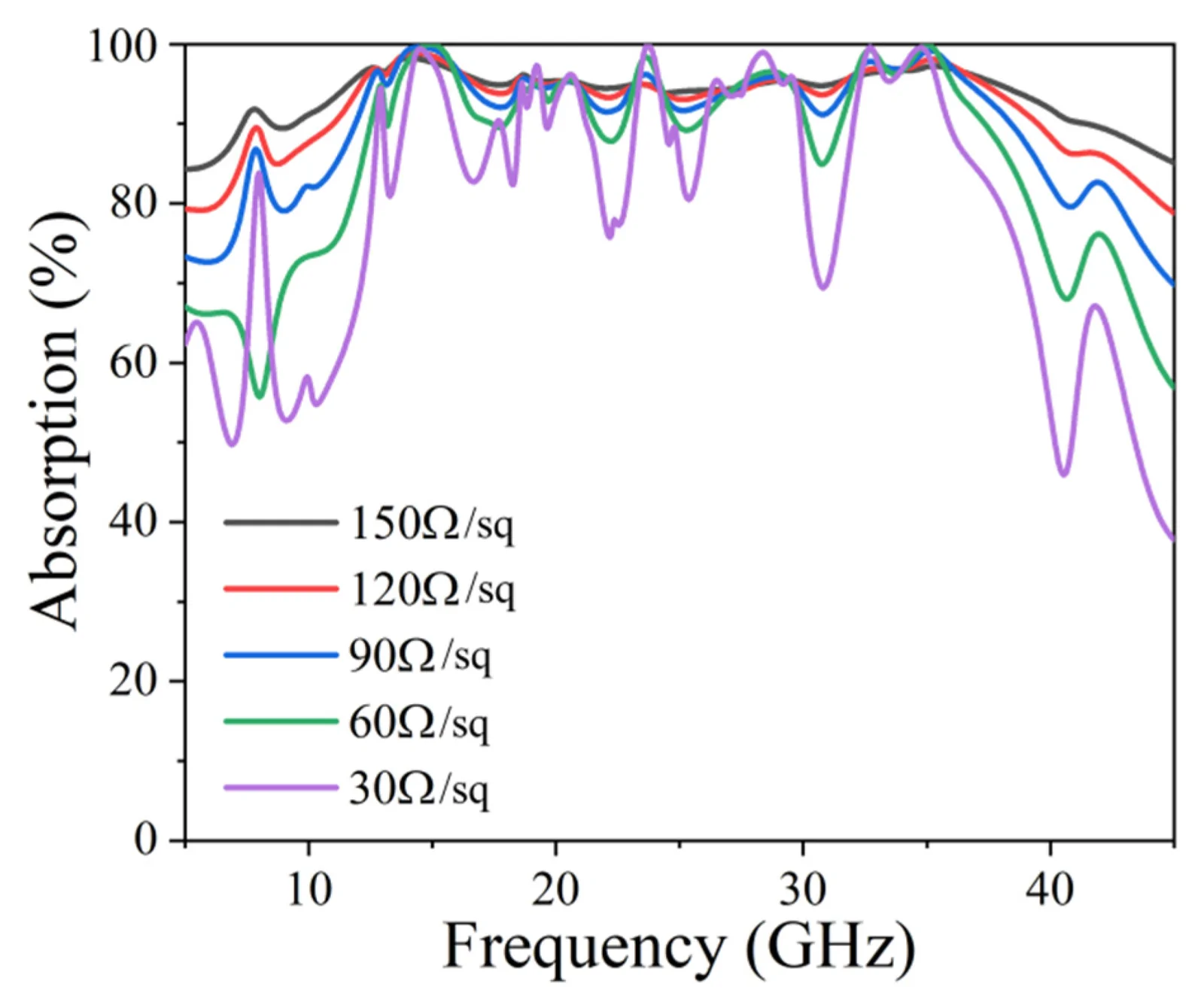
Simulated absorption spectra of the metasurface with different ITO sheet resistances.

The sheet resistance of the ITO films is a critical parameter for tuning the absorption performance. To investigate its influence, we performed simulations with five representative sheet resistances: 30, 60, 90, 120, and 150 Ω/sq, with the corresponding absorption spectra shown in Figure 6. The optimal broadband absorption is achieved at 150 Ω/sq, where the absorptance remains consistently high across the wide frequency range. As the sheet resistance decreases, the ohmic loss in the conductive films varies, leading to noticeable fluctuations in the absorption curves. At 30 Ω/sq, significant absorption dips appear in both the low- and high-frequency bands, substantially narrowing the effective absorption bandwidth. Excessively low sheet resistance results in insufficient ohmic loss to dissipate the incident electromagnetic energy efficiently.

**Figure 7:**
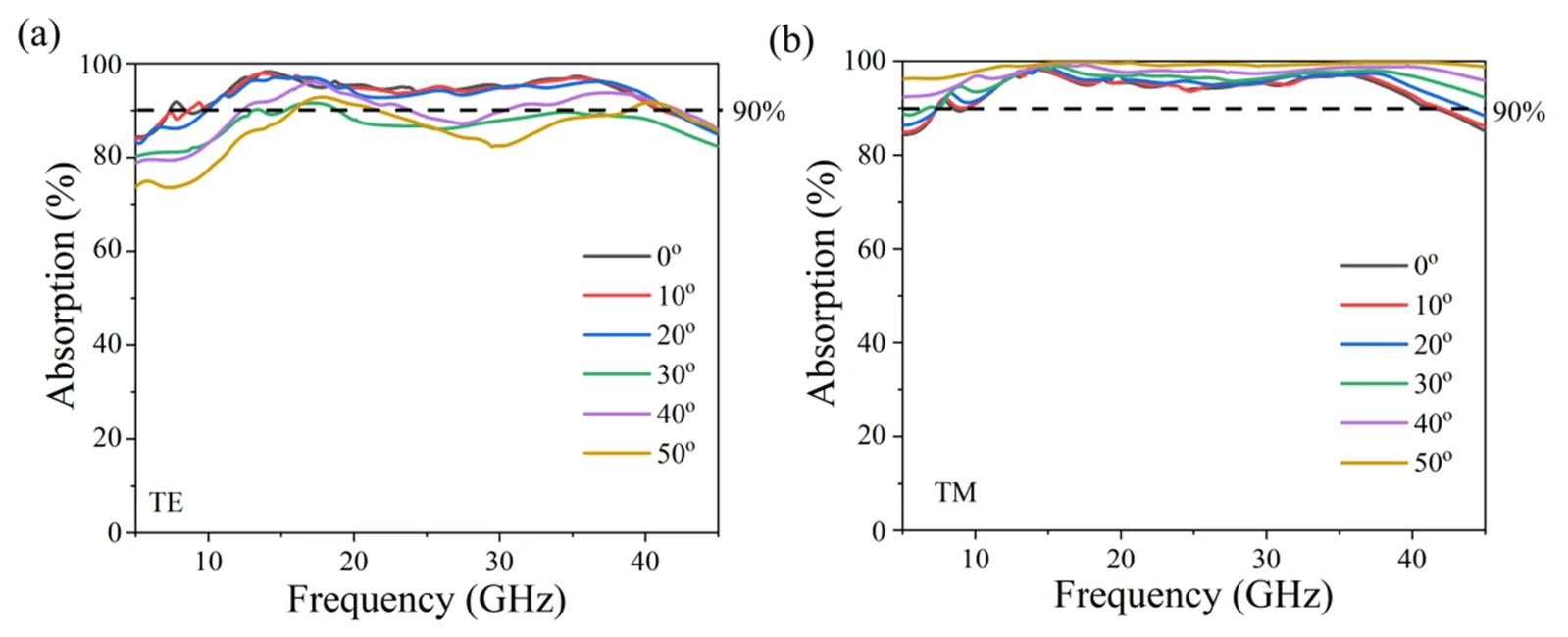
Absorption spectra of the metasurface under different incident angles: (**a**) TE polarization; (**b**) TM polarization.

To evaluate the performance stability under oblique incidence, we performed simulations for both TE and TM polarizations with incident angles ranging from 0° to 50°, and the corresponding absorption spectra are shown in Figure 7. For TM polarization, the absorptance remains above 90% over a broad frequency band even at an incident angle of 50°, demonstrating good angular tolerance. For TE polarization, however, as the incident angle increases, the absorption level decreases in both the low- and high-frequency regions, resulting in a gradual narrowing of the effective bandwidth. Overall, the proposed multilayer stacked metasurface exhibits superior angular insensitivity for TM-polarized waves compared to TE-polarized waves, maintaining good broadband absorption performance over a wide range of incident angles. The wide-angle absorption performance mainly arises from two structural features. First, the multilayer stacked configuration forms a smooth impedance gradient from free space to the absorbing structure, which effectively alleviates impedance mismatch under oblique incidence. Second, the symmetric subwavelength unit cell excites resonant modes that are weakly sensitive to incident angles, especially for TM polarization.

## 3. Fabrication and Experimental Measurements

**Figure 8:**
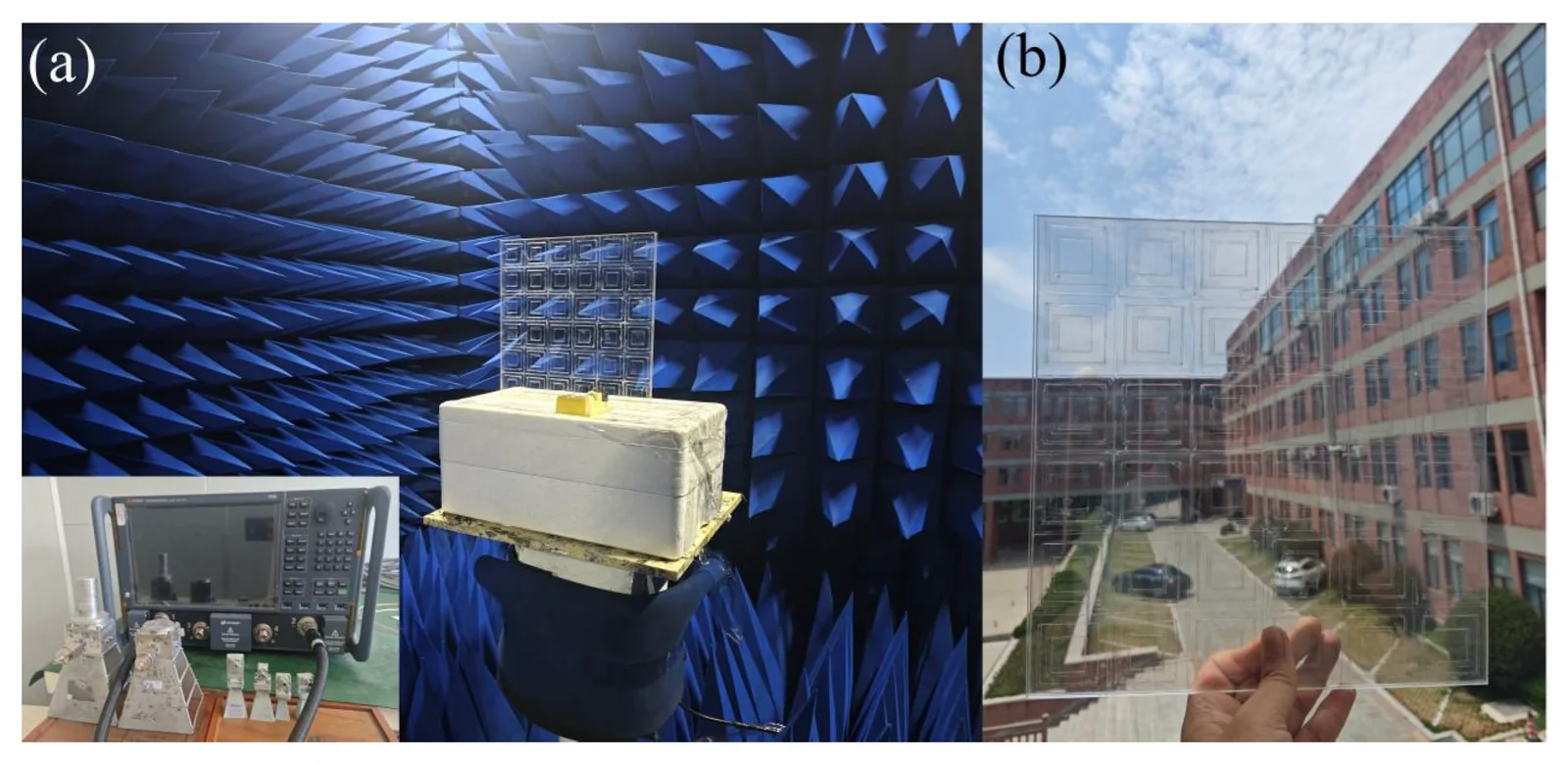
Fabricated sample and experimental setup: (**a**) microwave anechoic chamber measurement configuration; (**b**) photograph demonstrating the optical transparency of the metasurface sample.

**Figure 9:**
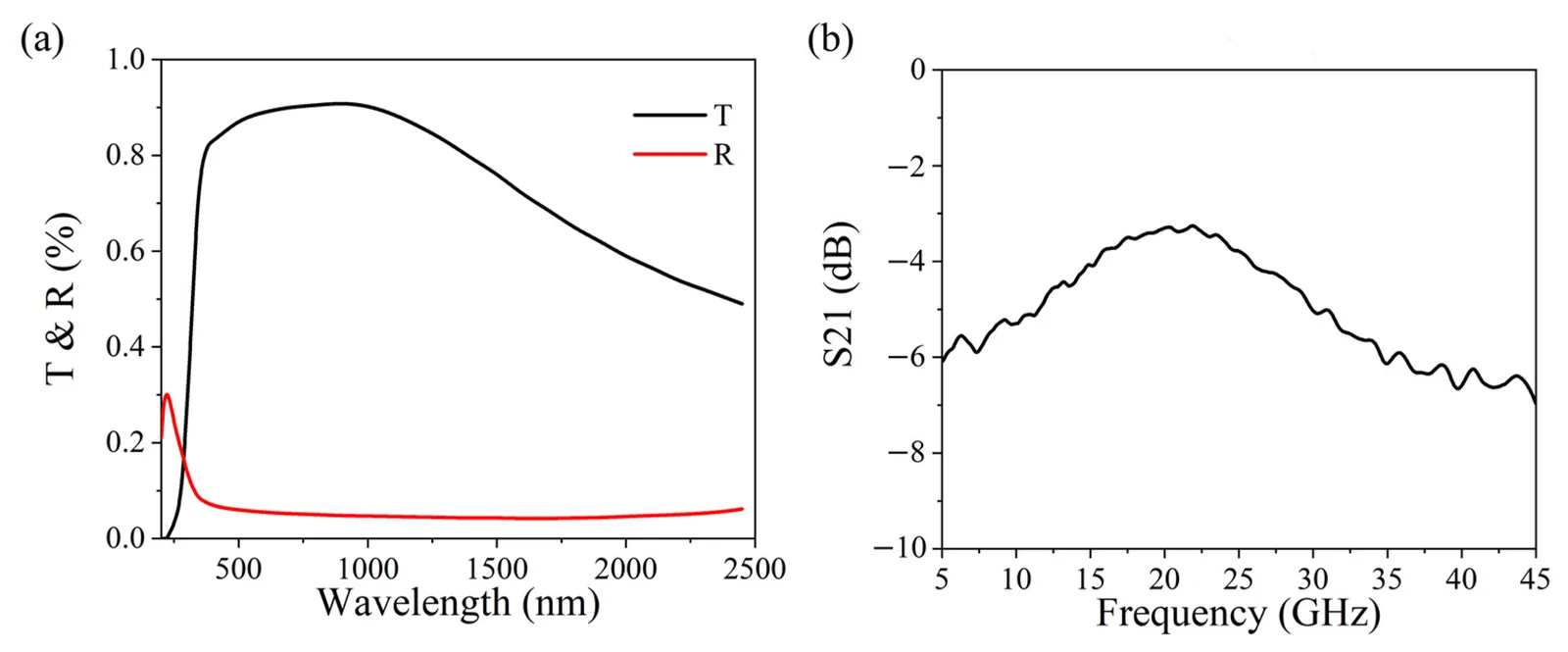
Measured properties of the single-layer ITO film. (**a**) Transmittance (T) and reflectance (R) spectra in the visible-near-infrared range for evaluating optical transparency; (**b**) measured microwave transmission coefficient (S21), which characterizes the microwave loss and shielding performance of bare ITO film.

Based on the optimized structural parameters, we fabricated a prototype of the optically transparent microwave absorbing metasurface, consisting of a 6 × 6 periodic array of unit cells. The measurement scenario is depicted in Figure 8a, where the sample is mounted on a fixture and the entire system is placed inside the chamber lined with absorbing pyramids, effectively eliminating spurious reflections from the environment. Figure 8b visually demonstrates the optical transparency of the sample, with the building behind clearly visible through the metasurface panel. This confirms that the multilayered ITO composite structure simultaneously possesses microwave absorption and optical transmission capabilities. The ITO films used in this work are standard commercial products from Xingyuan Technology, with key parameters listed in Table 1. Figure 9 presents the measured optical and electromagnetic properties of the single-layer ITO film. As depicted in Figure 9a, the film features high transmittance and low reflectance over the visible to near-infrared spectral range, which guarantees favorable optical transparency of the proposed multilayer absorbing metasurface. As shown in Figure 9b, the measured microwave transmission coefficient (S21) exhibits distinct electromagnetic attenuation across the investigated frequency band, confirming that the resistive ITO film provides adequate ohmic loss for microwave energy dissipation.

Since the bottom layer is a 150 Ω/sq resistive ITO film rather than an opaque metallic ground plane, the transmitted power cannot be neglected. The absorptance is calculated as A = 1 − R − T for both simulation and measurement, where R and T denote the power reflectance and transmittance, respectively. All measurements were performed in a standard microwave anechoic chamber using a Keysight N5225B vector network analyzer with three pairs of standard-gain horn antennas covering 2–18 GHz, 18–26.5 GHz and 26.5–45 GHz. For reflection measurement, the co-located transmitting/receiving antennas were placed 4 m from the sample along the surface normal; for transmission measurement, the two antennas were coaxially arranged on opposite sides of the sample, each 2 m away, satisfying the far-field condition for the 6 × 6 array. A comparative calibration method was applied: reflection was calibrated against a metal plate of identical size, and transmission was calibrated against free space. No time-gating was used, as the anechoic chamber sufficiently suppresses background multipath interference. Regarding higher-order Floquet modes, the 50 mm unit cell period theoretically supports diffracted orders above ~6 GHz; however, our antennas are aligned strictly along the sample normal and only collect zero-order specular reflection and forward transmission, consistent with the standard measurement convention in this field.

**Figure 10:**
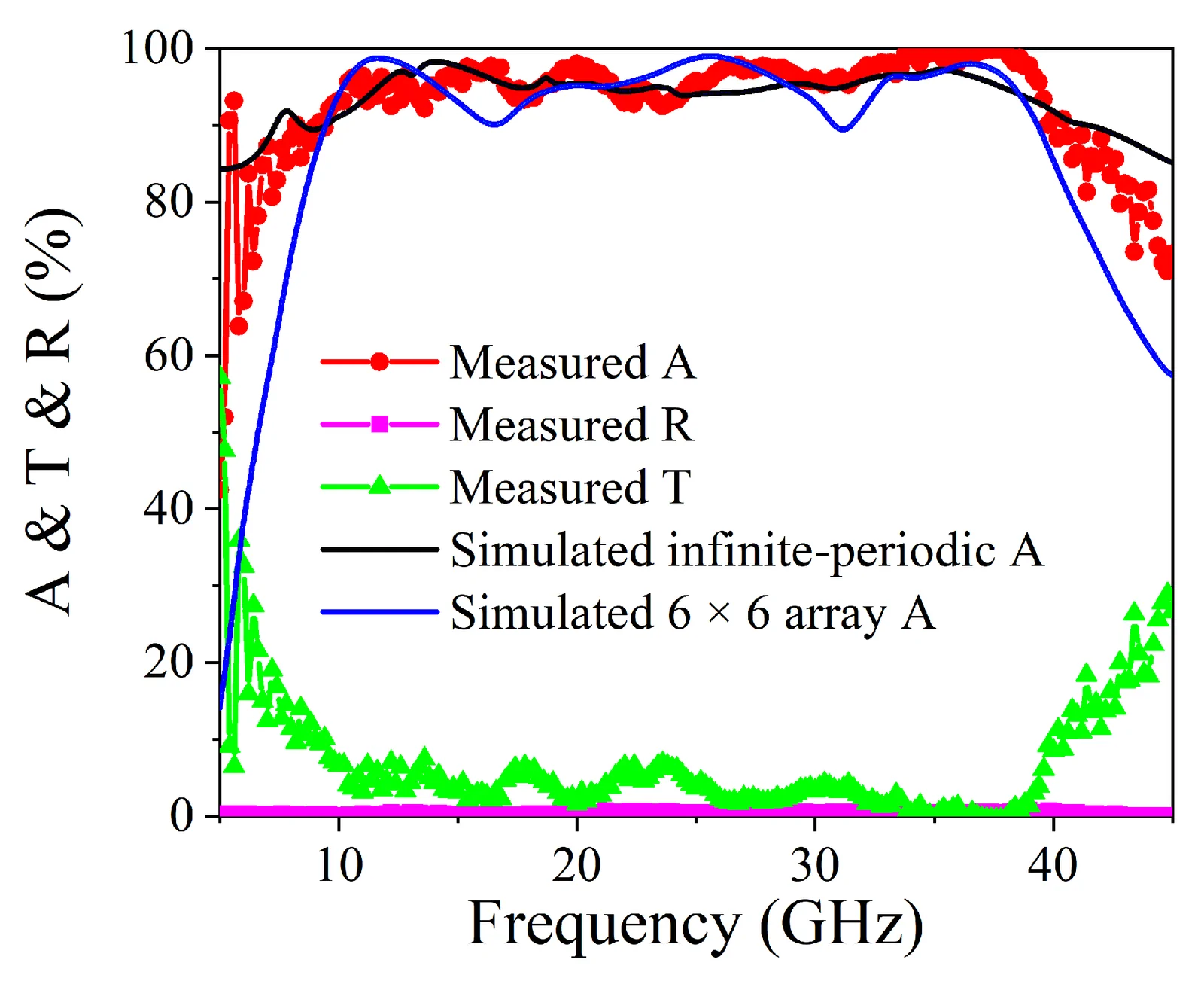
Simulated and measured power absorptance, reflectance and transmittance of the proposed metasurface. The black and blue lines represent the simulated absorptance of the infinite-periodic structure and the 6 × 6 finite array, respectively. The red, magenta and green dotted lines denote the measured absorptance, transmittance and reflectance, respectively.

Figure 10 presents the simulated and measured power absorptance of the proposed metasurface, together with the experimentally extracted reflectance and transmittance. Both the measured reflectance and transmittance remain at low levels across the effective absorption band. The absorptance calculated via A = 1 − R − T shows good agreement with the numerical simulation.

As shown in the Figure 10, the absorption trend and 90% bandwidth of the 6×6 finite array agree closely with the infinite-periodic model. The slight deviation is primarily attributable to the truncation effect, where the peripheral unit cells experience a strongly asymmetric electromagnetic environment and induce edge diffraction. In contrast, the majority of cells in the central region remain under near-periodic boundary conditions and thus govern the overall response. Simulations of the infinite periodic structure show that the absorptance exceeds 90% in the frequency range of 9.5–41.3 GHz, corresponding to an effective absorption bandwidth of 31.80 GHz and a fractional bandwidth of 125.2%. For the 6×6 finite array, the simulated 90% absorption band spans 9.4–39.2 GHz, with a fractional bandwidth of 121.0%. The measured absorptance remains above 90% from 9.2 to 40.2 GHz, yielding an effective bandwidth of 31.0 GHz and a fractional bandwidth of 125.5%. Overall, the measured absorption spectrum agrees well with the simulated curve in terms of both trend and the high-absorption band, thereby validating the proposed design. The minor discrepancies between the measured and simulated results can be attributed to fabrication tolerances, lamination gaps between the multilayer films, differences between the actual material electromagnetic parameters and the simulation inputs, the intrinsic dielectric loss of the constituent materials, as well as weak residual clutter in the measurement environment. These comparisons confirm that the fabricated optically transparent metasurface achieves ultra-wideband efficient microwave absorption, with the simulation results effectively supported by experimental data.

**Figure 11:**
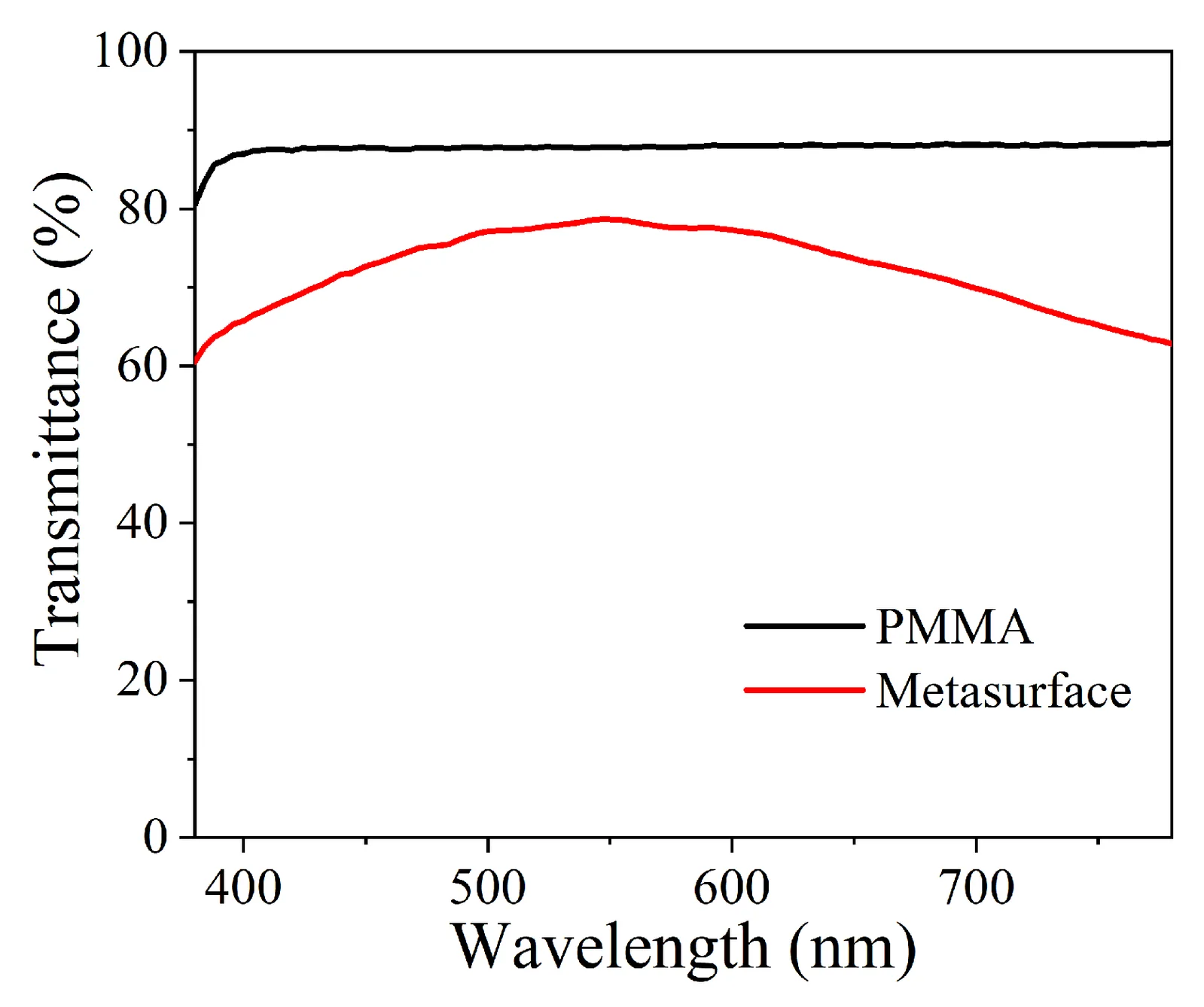
Measured optical transmittance spectra of the proposed metasurface and the reference multilayer PMMA slab in the visible wavelength range of 380–780 nm.

To quantitatively verify the optical transparency of the proposed metasurface, we characterized the optical transmittance of the fabricated prototype across the visible wavelength range (380–780 nm) using a PerkinElmer LAMBDA 1050 UV-Vis-NIR spectrophotometer (PerkinElmer, Shelton, CT, USA). A pure multilayer PMMA stack with the same total thickness was also measured as a reference. As presented in Figure 11, the reference PMMA stack achieves an average transmittance above 90% in the visible band. After integrating four patterned ITO resonant layers, the metasurface still retains transmittance higher than 60% over the entire 380–780 nm range. The moderate decrease in transmittance mainly arises from the intrinsic reflection and absorption of the ITO conductive films, which is consistent with the well-recognized optical properties of ITO materials. This result quantitatively confirms that the proposed metasurface simultaneously possesses ultra-wideband microwave absorption capability and good optical transparency.

**Figure 12:**
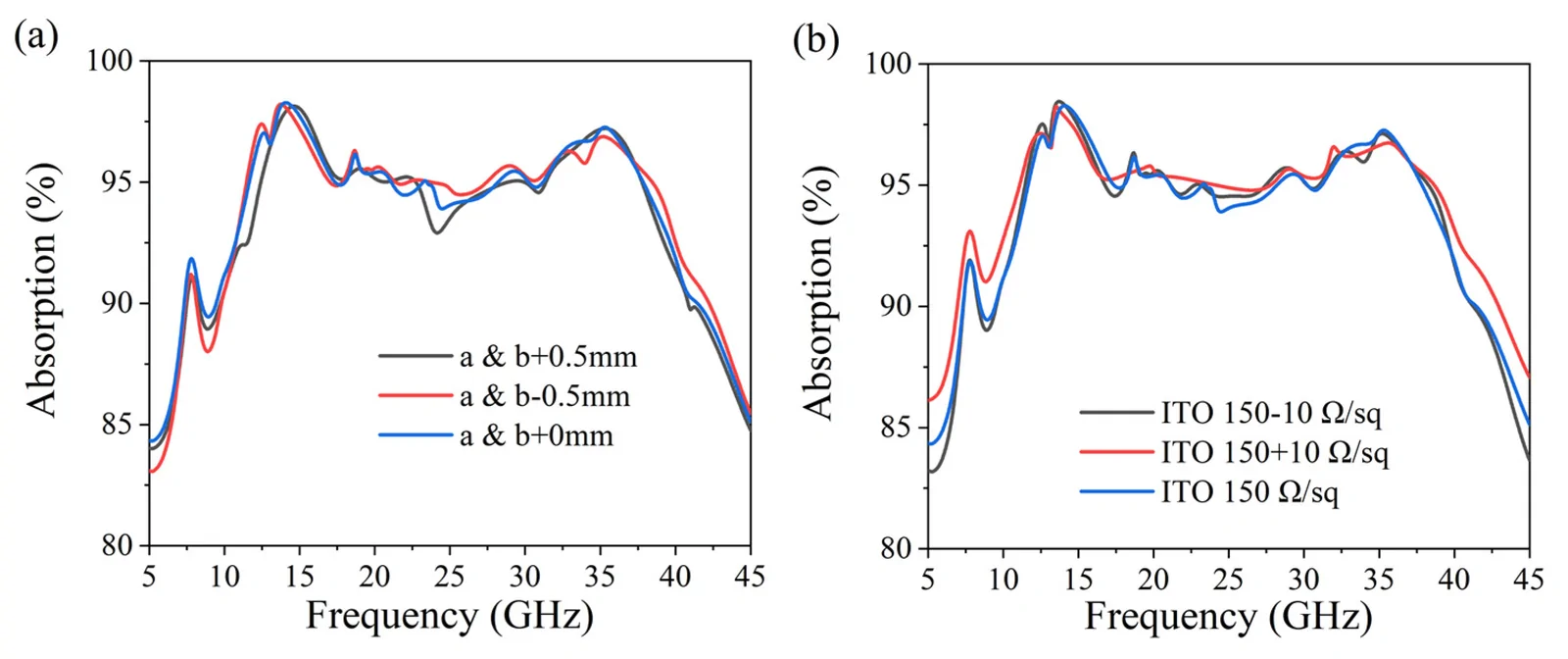
Fabrication tolerance analysis of the proposed absorber. (**a**) Absorption spectra under ±0.5 mm dimensional deviations of ITO patterns; (**b**) absorption spectra under ±10 Ω/sq variations of ITO sheet resistance.

To investigate the fabrication tolerance of the proposed absorber, parametric analyses of geometric dimension and sheet resistance deviations are carried out, as shown in Figure 12. Figure 12a presents the absorption spectra with ±0.5 mm dimensional deviations of all ITO pattern parameters (*a*_1_, *a*_2_, *a*_3_, *a*_4_, *b*_1_, *b*_2_) from the nominal values. Figure 12b depicts the absorption performance under ±10 Ω/sq variations of ITO sheet resistance relative to the designed value of 150 Ω/sq. The results indicate that both types of deviations cause negligible degradation to the absorption performance, and the 90% absorption bandwidth remains basically unchanged within the typical processing accuracy range, demonstrating favorable fabrication robustness of the proposed structure.

## 4. Discussion and Conclusions

Mechanism analysis reveals that the low-frequency (14.0 GHz) resonance involves all four ITO layers with strong interlayer coupling, whereas the high-frequency (35.2 GHz) resonance is governed by the upper three ITO layers with a standing-wave magnetic field distribution. Independent resonant modes at different frequencies superimpose to form continuous broadband absorption. Sheet resistance study indicates optimal impedance matching at 150 Ω/sq; lower resistance leads to insufficient ohmic loss and pronounced absorption dips. For oblique incidence, absorption remains stable up to 50° under TM polarization, while the bandwidth slightly contracts at large angles for TE polarization. Limitations: TE performance at large angles could be improved by anisotropic structures or symmetry optimization; current experimental verification is on a 6 × 6 sample, and large-area fabrication reliability needs further study; the total thickness (~6.4 mm) might be reduced using high-permittivity dielectric materials without degrading electrical performance. Table 2 summarizes the performance of representative ITO-based microwave absorbing structures. The proposed design achieves a 90% absorption relative bandwidth of 125.5% while maintaining optical transparency, showing a clear bandwidth advantage over most transparent absorbing counterparts. Although its total thickness is slightly larger than that of some single-layer or narrowband structures, it adopts simple square patch and square ring patterns that can be fabricated by mechanical cutting of commercial ITO films, striking a favorable balance between broadband absorption performance and fabrication feasibility. Future work will explore whether parameter optimization can yield pattern-free designs, or compress the current multi-layer stack into a single-layer configuration. We aim to achieve broadband absorption with improved performance with more simplified structures.

In summary, we have proposed and validated an optically transparent ultra-wideband microwave absorbing metasurface based on multilayer ITO films. The structure employs three PMMA dielectric layers and four ITO conductive layers (top patch, two intermediate square rings, and bottom continuous film) in a stacked configuration, overcoming the trade-off between bandwidth and transmittance through multilayer resonant coupling. The optimized design exhibits an absorptance above 90% over 9.50–41.30 GHz with a fractional bandwidth of 125.2% in simulation, while the fabricated sample achieves an absorptance above 90% over 9.2–40.2 GHz with a fractional bandwidth of 125.5% in measurement, showing good agreement between simulation and experiment. The sample also demonstrates good optical transparency. This multilayer stacked transparent absorbing metasurface, featuring both ultra-wideband microwave absorption and optical transparency, holds potential for applications in optoelectronics-compatible stealth, transparent electromagnetic shielding, and smart windows.

## Figures and Tables

**Table 1:** Key parameters of the commercial ITO films used for sample fabrication.

Model Number	Substrate	Sheet Resistance	Substrate Thickness	Visible Transmittance
SMH125H-150Y	PET	150 Ω/sq	0.125 mm	≥85%

**Table 2:** Performance Comparison with ITO absorber.

Ref.	Materials	Structure	Absorption Frequency (>90%)	Fractional Bandwidth	Thickness	Optical Transparency
[24]	ITO + PMMA	Multilayer + FSS structure	6.6–18.0 GHz	92.70%	4.525 mm	Yes
[25]	ITO + PET	Multilayer structure with cross-shaped patches	8.6–75.8 GHz	159%	3.55 mm	No
[27]	ITO + Si/SiO_2_	Salisbury screen + metamaterial	7.4–8.2 GHz	10.20%	2.0 mm	No
[28]	ITO + PET + PDMS	Square ring array	6.2–19.4 GHz	103%	3.3 mm	Yes
[29]	ITO + PVC + PET	Binary coding structure	5.3–15 GHz	95.60%	4.3 mm	Yes
[30]	ITO + Au + SiO_2_	SRR array	1530–2700 nm	55.30%	300 nm	No
This work	ITO + PET + PMMA	Square patch + square ring	9.2–40.2 GHz	125.5%	6.5 mm	Yes

## Data Availability

The raw data supporting the conclusions of this article will be made available by the authors on request.
